# Overlapping anti-*N*-methyl-d-aspartate receptor (NMDAR) encephalitis with neuromyelitis optica spectrum disorders: a case report

**DOI:** 10.1186/s40001-022-00697-w

**Published:** 2022-05-09

**Authors:** Jialin Pan, Begench Ovlyakulov, Lili Zhou

**Affiliations:** 1 Department of Neurology, The Second Affiliated Hospital and Yuying Children’s Hospital of Wenzhou Medical University, Zhejiang Province, Wenzhou, 325027 China; 2 Department of Clinical Medicine, The Second Affiliated Hospital and Yuying Children’s Hospital of Wenzhou Medical University, Zhejiang Province, Wenzhou, 325037 China

**Keywords:** Case report, Anti-NMDAR encephalitis, NMOSD, Overlapping syndrome

## Abstract

**Background:**

Anti-*N*-methyl-d-aspartate receptor (NMDAR) encephalitis can coexist with neuromyelitis optica spectrum disorder (NMOSD). Patients with overlapping anti-NMDAR encephalitis with positive NMDAR antibodies and aquaporin 4 immunoglobulin G (AQP4-IgG)-seropositive NMOSD are rare but should not be ignored.

**Case presentation:**

This report describes a unique case of anti-NMDAR encephalitis coexisting with NMOSD. A 27-year-old male presented with blurred vision, cognitive impairment, psychosis, dysphagia, gait instability and urinary incontinence. Brain magnetic resonance imaging (MRI) showed abnormal signals in the right cerebellar hemisphere, temporal lobe, and corpus callosum. NMDAR antibodies were positive in the CSF. AQP4-IgG antibodies were positive in the serum. The patient's condition was stable following intravenous gamma globulin, corticosteroids, immunosuppressants and symptomatic treatments.

**Conclusions:**

This case provides further evidence for the occurrence of anti-NMDAR encephalitis overlapping NMOSD with AQP4-IgG-seropositive in a Chinese patient. However, the mechanisms underlying the occurrence of double-positive antibodies remain elusive.

## Introduction

Autoimmune encephalitis is considered one of the most important causes of non-infectious acute encephalitis. It was reported that the frequency of anti-*N*-methyl-d-aspartate receptor (NMDAR) encephalitis exceeds that of individual viral etiologies in young individuals [1], less than 30 years of age. The disease predominantly affects children and young female patients, and its onset can be acute, subacute or may become chronic. Patients with anti-NMDAR encephalitis frequently present with behavioral complaints, psychosis, movement disorders (e.g., orofacial dyskinesia, dystonia) and seizures, and most related with ovarian teratomas [2]. Neuromyelitis optica spectrum disorder (NMOSD) is an inflammatory disorder of the central nervous system that typically presents with clinical signs of optic nerve damage and longitudinally extensive transverse myelitis [3]. The core clinical characteristics required to diagnose patients with NMOSD with AQP4-IgG include clinical syndromes or MRI findings related to the optic nerve, spinal cord, area postrema, another brainstem, diencephalic or cerebral presentations [4]. In addition, the presence of AQP4 antibody is disease specific to neuromyelitis optica if tested by proper assay methods. In this case report, we present a young male with two rare overlapping autoimmune syndromes; anti-NMDAR encephalitis and NMOSD. We believe that this case is rare and clinically challenging because of the two overlapping syndromes and the fast recovery.

## Case presentation

A previously healthy 22-year-old male Han Chinese presented in June 2019 with a 10-day history of epilepsy, gait instability, urinary incontinence. One year ago, the patient had suddenly blurring of vision, cognitive impairment, a rapid verbal reduction and psychiatric behaviors. Previously he was diagnosed with schizophrenia in Hangzhou Seventh People's Hospital (The Mental Health Center Affiliated Zhejiang University School of Medicine) and given some antipsychotic drugs and electroconvulsive therapy eight times. Unfortunately, his symptoms were not relieved; furthermore, they worsened. His neurological exam revealed abnormal higher mental function, mild visual impairment, mild ataxia, urinary incontinence. Motor and sensory examination was normal, and deep tendon reflex were+2 in all limbs.

**Fig. 1:**
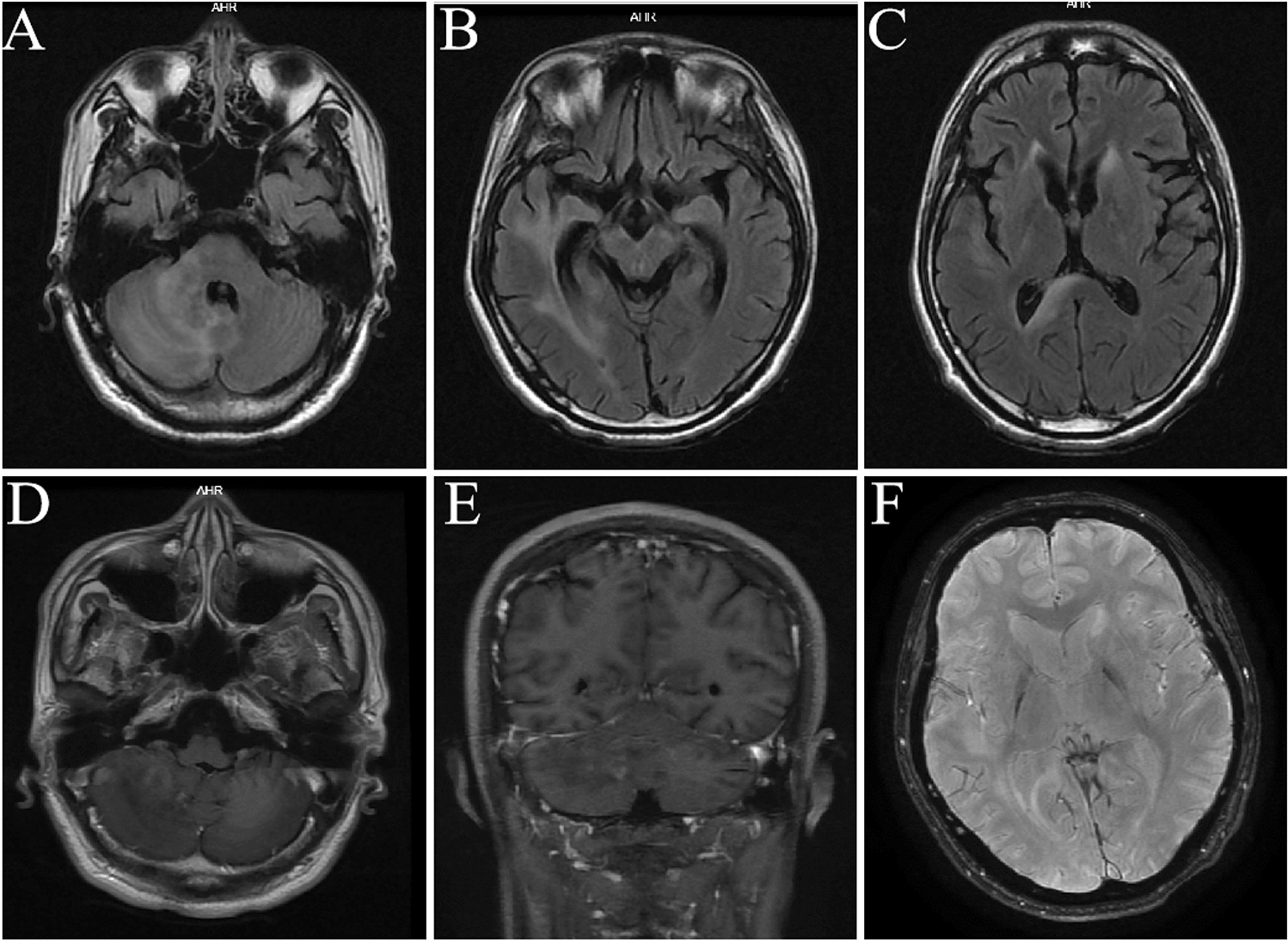
Axial T2-weighted FLAIR head MRI shows hyper-intense lesions in the right cerebellar hemisphere, brain stem, temporal lobe, and corpus callosum (**A**–**C**). Axial T1-weighted image show lesions in the right cerebellum were punctate enhancing after gadolinium administration (**D**, **E**). SWI scan was normal (**F**)

Subsequently, brain MRI on fluid-attenuated inversion recovery (FLAIR) and diffusion images revealed hyper-intense lesions in right cerebellar hemisphere, temporal lobe, including corpus callosum, lesions in the right cerebellum were punctate enhancing with gadolinium contrast (Fig. 1). Cervical and thoracic MRI was normal. FDG-PET/CT imaging showed that the FDG metabolism of bilateral frontal, temporal, midbrain and cerebellum was reduced; the temporal and cerebellum atrophy obviously (due to the FDG-PET/CT was done in other hospitals, unfortunately we only got a report). Conventional electroencephalography (EEG) showed there were several θ waves and sharp waves on both sides. Unfortunately, due to the patient's abnormal behavior, the visual evoked potential was not taken successfully. Regrettably, an orbital MRI was not done.

Following lumbar puncture examination, the pressure was 148 mmH2O, CSF analysis showed elevated protein of 856 mg/L (NR 120–600 mg/l) with normal glucose level and WBC count was 0 (NR 0–8 × 10^6^/l). Oligoclonal bands were not detected in the CSF (A type I profile, IgG intrathecal synthesis rate, 8.49 mg/24 h, IgG index 0.7, NR < 0.680). Serum and CSF samples were sent to test for presence of both anti-NMDAR antibodies and NMOSD antibodies.

The results confirmed the diagnosis of both anti-NMDAR encephalitis and NMOSD. Anti-AQP4 IgG was detected in the serum samples at a titer of 69.65 U/ml (normal value ≦ 5U/ml), 3.67 U/ml in the CSF samples, using enzyme linked immunosorbent assay (ELISA). Moreover, anti-NMDAR antibodies were detected in the CSF at a titer of 1:10, using the cell-based indirect immunofluorescence assay (CBA). Serum was not examined for the presence of anti-NMDAR antibodies. The patient tested negative for anti-myelin oligodendrocyte glycoprotein (MOG) antibodies, glial fibrillary acidic protein (GFAP), as well as other autoimmune encephalitis antibodies (anti-GABAB, anti-AMPAR1, anti-AMPAR2, anti-LGI1 and anti-CASPR2). The antinuclear antibodies (ANA), anti-neutrophil cytoplasmic antibodies (ANCA), anti-Sjögren syndrome A (SSA) antibody and anti-Sjögren syndrome B (SSB) antibodies were also negative.

The autoantibodies assay results supported the diagnosis of overlapped anti-NMDAR encephalitis and anti-AQP4 NMOSD for our patient. The differential diagnosis, at that time, was consistent with viral encephalitis, acute disseminated encephalomyelitis (ADEM). However, CSF study and MRI findings made viral encephalitis and ADEM unlikely.

**Fig. 2:**
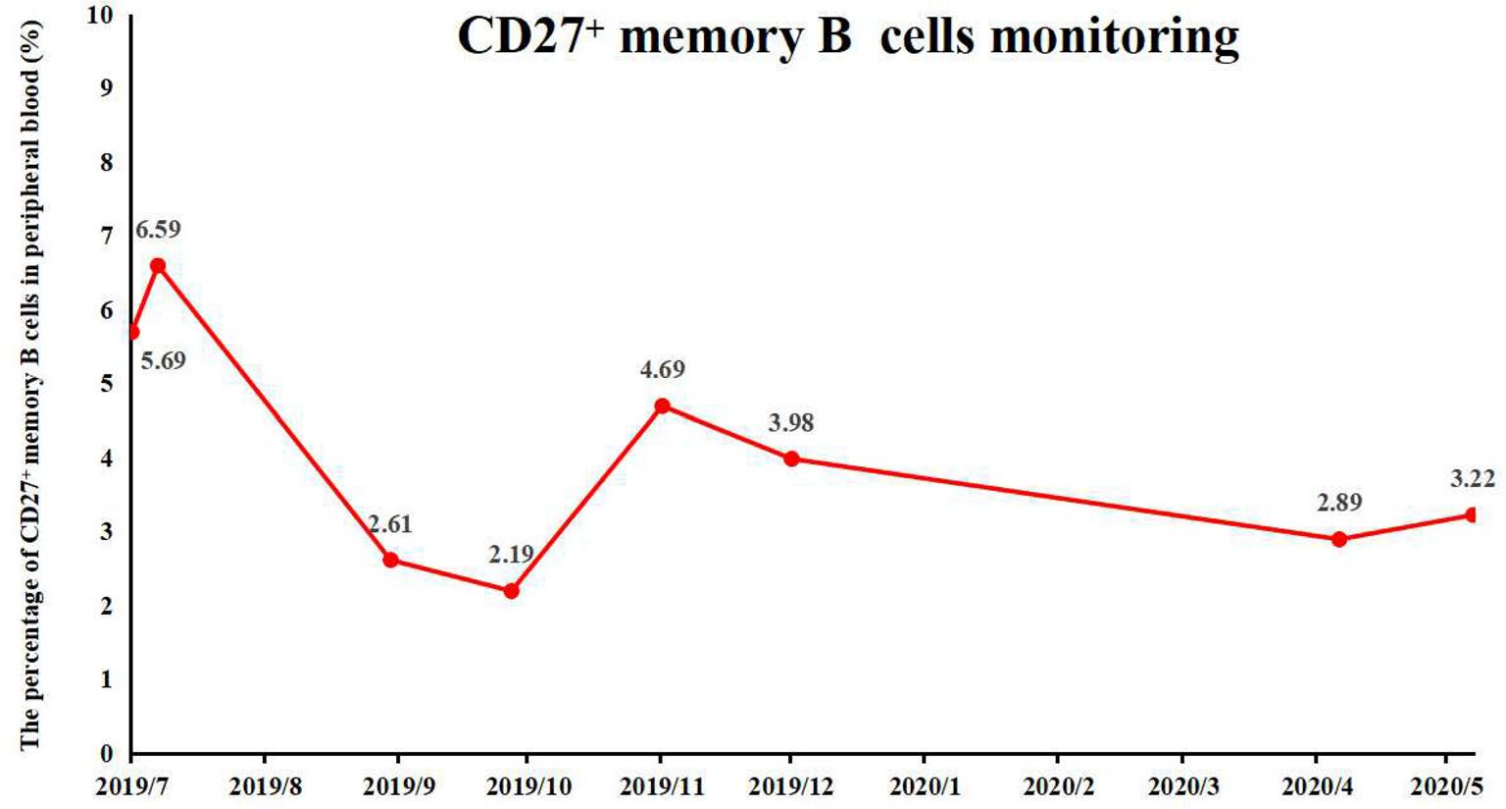
The percentage of CD27^+^ memory B cells in peripheral blood was monitored several months, which prompt that the patient's condition was well controlled after immune therapy

**Fig. 3:**
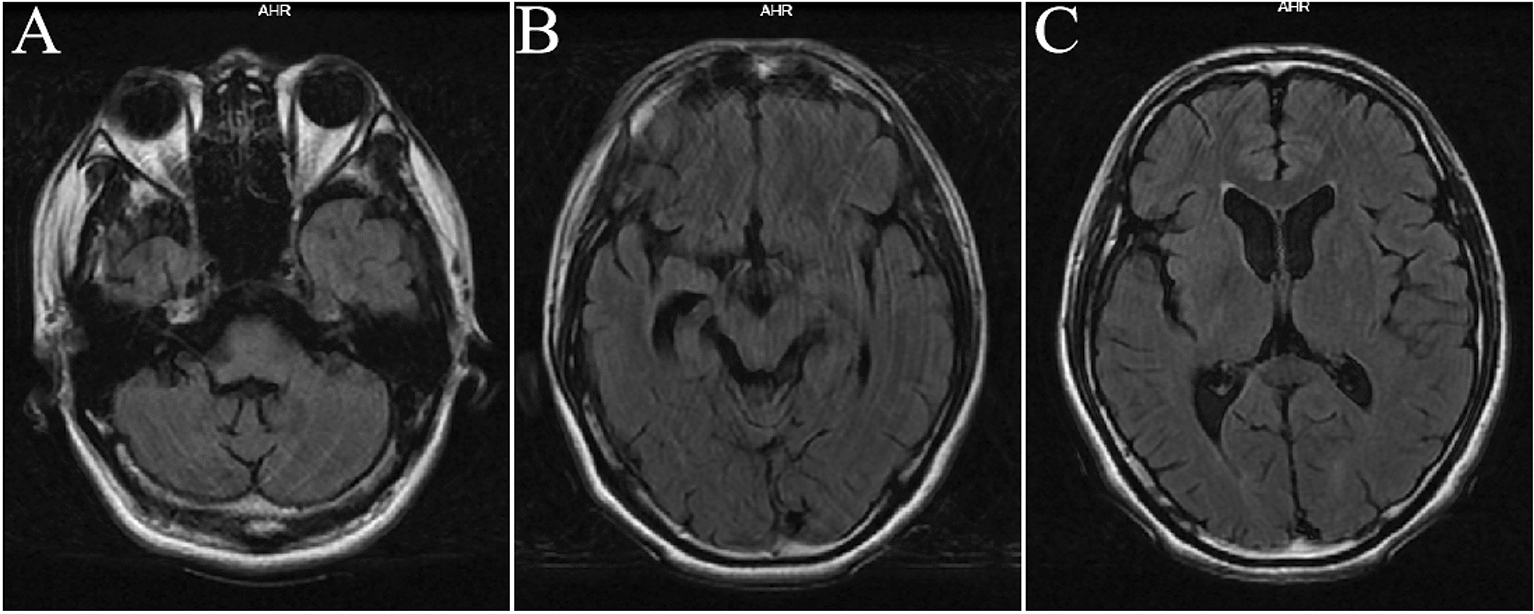
Axial T2-weighted head MRI showing hippocampus and midbrain atrophy, lateral ventricles and the third ventricle enlargement

The patient was treated with intravenous methylprednisolone at a dose of 1 g once daily for 5 days and subsequently received IVIG 0.4 g/kg/dose once daily for 5 days. After which, the corticoid dose was gradually reduced to 50 mg orally and started with azathioprine 50 mg bid daily to prevent further relapses. Following the immune therapy, life support, and symptomatic treatment, the patient's symptoms were recovered partly after 14 days. He was scheduled for regular follow-ups, and he is stable with no relapse so far. Now he has recovered well and back to his normal baseline daily activities, with a little memory disorder and mild visual impairment, no more epilepsy onset. The percentage of CD27^+^ memory B cells in peripheral blood was monitored several months, which prompt that the patient's condition was well controlled after immune therapy (Fig. 2). The brain MRI has been taken after 2 years of follow-ups, which found no obvious abnormal signals in the brain, but the ventricles and cistern brain sulci were enlarged (Fig. 3).

## Discussion

We describe a case of a male Chinese patient who presented simultaneously with two distinct autoimmune diseases: anti-NMDAR encephalitis with positive NMDAR antibodies in the CSF and NMOSD with AQP4-IgG-seropositivity. The patient had core clinical characteristics, including psychiatric behavior, cognitive impairment and verbal reduction, with NMDAR antibody was present in CSF. Thus, the patient was diagnosed with anti-NMDAR encephalitis. However, two aspects of his symptoms were inconsistent with anti-NMDAR encephalitis. Firstly, the patient had a history of optic neuritis. Secondly, the AQP4 antibody was positive in the serum. Therefore, definitive diagnosis of NMOSD can be made in the presence of one of the six major symptoms, and following the reasonable exclusion of other disorders.

Anti-NMDAR encephalitis is an immune-mediated disorder characterized by a complex neuropsychiatric symptom and by the presence of antibodies against the NMDAR subunit of GluN1 [5]. An underlying neoplasm is found in 25 to 40% of anti-NMDAR encephalitis patients, and usually 90% of the cases associated with ovarian teratoma, which more frequently occur in young females [6]. This strong association suggests a role of the tumor in the immunopathogenesis of the autoimmune disease. Furthermore, anti-NMDAR encephalitis has been reported in association with the central nervous system demyelinating diseases, such as ADEM (acute disseminated encephalomyelitis) [7], MOG-antibody related disease [8, 9] and NMOSD [10–12]. Also, the occurrence of atypical symptoms for NMOSD (e.g., psychiatric symptoms, neglect, and cognitive impairment) prompted to investigate further for evidence of other autoimmune diseases, such as anti-NMDAR antibodies encephalitis.

The pathogenesis of anti-NMDAR encephalitis may be attributed to antibody cross-linking and capping and internalization of the NMDARs, leading to decreased receptor density and the reduced synaptic function of the neurons [1]. The pathogenesis of NMOSD can result from astrocyte damage due to complement-dependent cytotoxicity mediated by AQP4 antibodies or demyelinating lesions due to T cell-mediated immunity [13]. Immune complexes can be form between the AQP4 and NR3A subunits of the NMDAR, suggesting that AQP4 may be involved in NMDAR-mediated signaling [14]. This suggests that AQP4 and NMDA may share functionality, but their correlation in patients with co-morbidity remains limited, the specific mechanisms of which now warrant further investigation.

Conventional treatment for anti-NMDAR encephalitis and NMOSD includes intravenous methylprednisolone, intravenous immunoglobulin (IVIG) and/or plasma exchange. Immunosuppressant is an essential and effective medicine used as a preventive therapy for both diseases. Our patient showed significant improvement with acute management (intravenous methylprednisolone and intravenous immunoglobulin—IVIG) and with the preventive therapy using azathioprine.

## Conclusion

The co-morbidity of anti-NMDAR encephalitis and NMOSD is less frequently reported. The specific mechanisms of double-positive antibodies remain elusive, and both the clinical features and imaging abnormalities of autoimmune neurological co-morbidities are superimposed, leading to difficulties during diagnosis. The early recognition, diagnosis and treatment of such an overlapping autoimmune diseases are the key factors in ensuring early and better recovery outcomes in the clinic.

## Data Availability

Data sharing is not applicable to this article as no datasets were generated or analyzed during the current study.
